# Development of prognostic signatures and risk index related to lipid metabolism in ccRCC

**DOI:** 10.3389/fonc.2024.1378095

**Published:** 2024-06-13

**Authors:** Wenbo Chen, Zhenyu Zhao, Hao Zhou, Shuang Dong, Xiaoyu Li, Sheng Hu, Shan Zhong, Ke Chen

**Affiliations:** ^1^ School of Basic Medical Sciences, Wuhan University, Wuhan, China; ^2^ Department of Urology, Tongji Hospital, Tongji Medical College, Huazhong University of Science and Technology, Wuhan, China; ^3^ Department of Hematology, Union Hospital, Tongji Medical College, Huazhong University of Science and Technology, Wuhan, China; ^4^ Department of Oncology, Hubei Cancer Hospital, Tongji Medical College, Huazhong University of Science and Technology, Wuhan, Hubei, China

**Keywords:** lipid metabolism, ccRCC, prognosis, immunotherapy, personalized treatment

## Abstract

**Background:**

Clear cell renal cell carcinoma (ccRCC) is a metabolic disorder characterized by abnormal lipid accumulation in the cytoplasm. Lipid metabolism-related genes may have important clinical significance for prognosis prediction and individualized treatment.

**Methods:**

We collected bulk and single-cell transcriptomic data of ccRCC and normal samples to identify key lipid metabolism-related prognostic signatures. qPCR was used to confirm the expression of signatures in cancer cell lines. Based on the identified signatures, we developed a lipid metabolism risk score (LMRS) as a risk index. We explored the potential application value of prognostic signatures and LMRS in precise treatment from multiple perspectives.

**Results:**

Through comprehensive analysis, we identified five lipid metabolism-related prognostic signatures (ACADM, ACAT1, ECHS1, HPGD, DGKZ). We developed a risk index LMRS, which was significantly associated with poor prognosis in patients. There was a significant correlation between LMRS and the infiltration levels of multiple immune cells. Patients with high LMRS may be more likely to respond to immunotherapy. The different LMRS groups were suitable for different anticancer drug treatment regimens.

**Conclusion:**

Prognostic signatures and LMRS we developed may be applied to the risk assessment of ccRCC patients, which may have potential guiding significance in the diagnosis and precise treatment of ccRCC patients.

## 1 Introduction

Renal cell carcinoma (RCC) represents the most prevalent form of kidney cancer, with 90% of cases being attributed to this type, and a higher incidence in males compared to females (1). Histologically, RCC can be categorized into various subtypes, among which clear cell renal cell carcinoma (ccRCC) is the most common, accounting for approximately 75% of cases (2). In contrast to other RCC subtypes, patients with ccRCC exhibit the lowest 5-year survival rates and are more prone to advanced T stage, metastatic disease, and higher-grade tumors (3, 4). Notably, ccRCC is characterized as a metabolic disease, with the formation of lipid droplets representing a distinct histological feature (5). In recent years, substantial advancements have been made in the treatment of ccRCC, encompassing nephrectomy, targeted therapies against vascular endothelial growth factor (VEGF), and emerging immunotherapeutic agents (6). However, the inherent toxicities of these therapies have limited their application, with generally poor overall response rates (7). Moreover, the presence of tumor heterogeneity underscores the need for personalized treatment strategies tailored to individual tumor characteristics (8, 9). Consequently, the exploration of novel prognostic signatures and the construction of corresponding risk indices related to tumor heterogeneity for prognosis prediction and precise therapy may hold significant clinical significance.

Tumor initiation relies on the reprogramming of cellular metabolism, as cancer cells undergo specific metabolic reprogramming to sustain cellular growth and proliferation (10). Most cancer types exploit lipids and cholesterol to meet their insatiable energy demands (11). ccRCC, a distinct subtype of RCC, is characterized by the accumulation of lipids within the cytoplasm. Research evidence has indicated the role of aberrant lipid accumulation in ccRCC disease progression (12, 13). Additionally, the inactivation of the AMPK-GATA3-ECHS1 pathway can induce fatty acid synthesis, promoting ccRCC growth (14). E2F1, through the activation of SREBP1-dependent fatty acid biosynthesis, facilitates the proliferation and metastasis of ccRCC (15). These lines of evidence collectively demonstrate the close association between lipid metabolism and the progression of ccRCC. Furthermore, the nutrient competition between tumor cells and immune cells within the tumor microenvironment results in various functional impairments of immune cells, subsequently affecting the efficacy of immunotherapeutic interventions (16). Research evidence suggests that interventions targeting the reprogramming of lipid metabolism can prevent effector T cell senescence within the tumor microenvironment and enhance the efficacy of tumor immunotherapy (17).

In this study, we evaluated the dysregulation and prognostic potential of lipid metabolism-related genes in ccRCC based on the bulk transcriptomic data from the Cancer Genome Atlas (TCGA) clear cell renal cell carcinoma (KIRC) cohort. Integrating single-cell transcriptome analysis, we ultimately identified a set of key lipid metabolism-related prognostic signatures. We explored the potential of prognostic signatures in predicting the occurrence of ccRCC and patient prognosis through machine learning model. Furthermore, based on these signatures, we developed a lipid metabolism risk score (LMRS) as a risk index in ccRCC and comprehensively analyzed the associations between LMRS and patient survival, patient genomic characteristics, immune cell infiltration, immunotherapy, and anticancer drug sensitivity. In summary, our study offers new insights into the potential application value of lipid metabolism in ccRCC.

## 2 Materials and methods

### 2.1 Data acquisition

In this study, clinical data and bulk transcriptomic data of 602 samples from the TCGA-KIRC cohort were downloaded from UCSC Xena (http://xena.ucsc.edu/, accessed on 2 January 2023) (18). The bulk transcriptomic data comprised the raw gene counts matrix and the corresponding fragments per kilobase of exon model per million mapped fragments (FPKM) matrix. The clinical data included sample pathology status, survival time, survival outcome, age, gender, and other relevant information. Additionally, microarray data from the GSE53757 (n=144) (19), GSE36895 (n=52) (20), GSE22541 (n=24) (21), and GSE67501 (n=11) (22) cohorts were obtained from the gene expression omnibus (GEO) database (https://www.ncbi.nlm.nih.gov/, accessed on 20 January 2023). The microarray data from E-MTAB-1980 (n=101) (23) were sourced from the EMBL-EBI database (https://www.ebi.ac.uk/, accessed on 12 February 2023). Sample information provided by data contributors was used to distinguish ccRCC samples and normal tissue samples in the GSE53757 and GSE36895 cohorts. Survival outcome information for ccRCC samples in the E-MTAB-1980 and GSE22541 cohorts was extracted from the corresponding supplementary files of the respective studies. The response data of 11 ccRCC patients to nivolumab in the immunotherapy cohort GSE67501 was provided by the authors. We retained only those samples in all cohorts that were explicitly labeled as ccRCC tumors or normal kidney tissues. Detailed baseline clinical data for all ccRCC cohorts are summarized in **Table 1**.

**Table 1:** Clinical characteristics of the samples in each study cohort.

Cohort	TCGA-KIRC	GSE53757	GSE36895	E-MTAB-1980	GSE22541	GSE67501
**Samples,n**	602	144	52	101	24	11
Tumor	531	72	29	101	24	11
Normal	71	72	23	–––	–––	–––
Age						
<60 years	276	–––	–––	41	–––	–––
>=60 years	326	–––	–––	60	–––	–––
Gender						
Male	399	–––	–––	77	–––	–––
Female	203	–––	–––	24	–––	–––
Vital status						
Alive	405	–––	–––	78	18	–––
Dead	197	–––	–––	23	6	–––
Response to immunotherapy						
Responsive	–––	–––	–––	–––	–––	4
Non-responsive	–––	–––	–––	–––	–––	7

'---' indicates that there is no information available for the corresponding entry in the table.

### 2.2 Transcriptomic data processing

The FPKM matrix from the TCGA-KIRC cohort was transformed into a transcripts per kilobase of exon model per million mapped reads (TPM) matrix for subsequent analyses, such as the evaluation of the level of immune cell infiltration. Specifically, for each gene within a sample, the FPKM value of that gene was divided by the sum of FPKM values for all genes within that sample. Subsequently, the library size of each sample was uniformly scaled to 1 000 000.

The transcriptomic data provided by the E-MTAB-1980 cohort was directly utilized for our analysis. Additionally, we downloaded microarray data from the GSE53757, GSE36895, GSE22541, and GSE67501 cohorts. We computed the signal intensities of each probe using the affy v1.74.0 package in R with the robust multichip average (RMA) algorithm. The GSE53757, GSE36895, and GSE22541 cohorts were all based on the GPL570 platform. We utilized the AnnoProbe v0.1.7 package in R to obtain annotations for the probes of the GPL570 platform and matched probe IDs with gene names. In cases where the same gene corresponded to multiple probes, the data from the probe with the highest signal intensity were retained as the final gene expression level. The GSE67501 cohort was based on the GPL14951 platform. We manually downloaded annotation information of the GPL14951 platform from GEO and employed the same method as mentioned above to convert the probe signal intensity matrix to a gene expression matrix. In addition, the microarray data from the Caki-1 and RPTEC/TERT1 cell lines in the GSE232951 dataset (GPL17692 platform) were also processed in the same manner as described above.

### 2.3 Differential gene expression analysis, functional enrichment analysis, and gene set enrichment analysis

We extracted normal control samples and matched KIRC samples from 71 patients in the TCGA-KIRC cohort. Using the raw gene counts matrix of the samples, we utilized the DESeq2 v1.36.0 (24) package in R to identify differentially expressed genes. Genes with | log2(FoldChange) | > 1 and an adjusted p-value< 0.05 were considered to have significantly changed expression levels. Pathway information was obtained from the kyoto encyclopedia of genes and genomes (KEGG) (https://www.kegg.jp, accessed on 5 March 2023) (25). We used the clusterProfiler v4.4.1 (26) package in R to perform gene functional enrichment analysis and gene set enrichment analysis (GSEA). Pathways with an adjusted p-value< 0.05 were considered significantly enriched in gene functional enrichment analysis. In the GSEA, we first sorted the genes according to their log2(FoldChange) from largest to smallest, and then carried out enrichment analysis. Visualization of the results from GSEA was completed using the enrichplot v1.16.1 package in R.

The differential gene expression analysis between Caki-1 and RPTEC/TERT1 cell lines was performed using limma v3.56.2.

### 2.4 Analysis of single-cell transcriptomic data

We obtained single-cell sequencing data of 7 ccRCC tumor samples and 6 normal samples in GSE159115 (accessed on 25 February 2023) (27) and incorporated them into the analysis pipeline using the Seurat v4.1.1 package in R (28). We retained only high-quality cells with more than 200 expressed genes and mitochondrial gene expression accounting for less than 10% of the total expression. The raw gene counts matrix was normalized using the NormalizeData() function with LogNormalize method. We then selected the top 2000 highly variable genes for principal component analysis (PCA) using the FindVariableFeatures(), ScaleData(), and RunPCA() functions. Batch differences between samples were eliminated using the harmony v0.1.1 package (29). The FindNeighbors(x, reduction = “harmony”) and FindClusters() functions were employed for cell clustering. Subsequently, the FindMarkers() and FindAllMarkers(x, min.pct = 0.3) function was used to identify differentially expressed genes in each cell cluster. Genes with | logfc.threshold | > 0.1 and an adjusted p-value< 0.05 were considered as differentially expressed genes. We obtained a list of marker genes for multiple cell types from Cell Taxonomy (https://ngdc.cncb.ac.cn/celltaxonomy) (30) and annotated cell types for each cell cluster based on the differentially expressed genes in each cell cluster. The uniform manifold approximation and projection method (UMAP) algorithm was then employed to generate two-dimensional mappings of all cells.

### 2.5 Estimation of single-cell metabolic levels

To study the activation levels of metabolic pathways within each cell, we utilized the scMetabolism v0.2.1 package (31) for evaluation. scMetabolism encapsulates multiple algorithms for quantifying metabolic activity at the single-cell resolution and is compatible with Seurat. We used the AUCell algorithm to assess the activation levels of KEGG metabolic pathways integrated within scMetabolism.

### 2.6 Cell culture

Caki-1 and RPTEC/TERT1 cell lines were purchased from the Cell Bank of Chinese Academy of Sciences (China). Caki-1 cells were cultured in McCOY’s 5A media (Sigma-Aldrich, USA) supplemented with 10% fetal bovine serum (HyClone, Logan, UT), 1% L-glutamine (Invitrogen, Carlsbad, CA), and 1% penicillin-streptomycin (Invitrogen, Carlsbad, CA), and incubated in 5% CO_2_ with a balance of air at 37°C. The RPTEC/TERT1 cells were cultured in DMEM/F12 medium (Invitrogen, Carlsbad, CA) containing 10% fetal bovine serum (HyClone, Logan, UT) and 1% L-glutamine (Invitrogen, Carlsbad, CA), and 1% penicillin-streptomycin (Invitrogen, Carlsbad, CA). Culture media were replaced every 2–3 days until the cells reached 80% confluence, and 0.25% trypsin was then added for cell passaging.

### 2.7 Quantitative RT-PCR

Total RNA was isolated from Caki-1 and RPTEC/TERT1 cells using RNAiso plus (Takara, 108–95-2). The RNA concentration and purity were determined through a NanoDrop One Spectrophotometer and 2100 Bioanalyzer (Agilent Technologies, Wilmington, DE, USA). A total of 500 ng RNA was reverse transcribed to cDNA and purified using the SuperScript First-Strand Synthesis System for RT-PCR (Invitrogen, 11904–018). Real-time RT-PCR was performed on the BioRAD CFX96™ Touch (Bio-Rad Laboratories Inc.), using SYBR Green Supermix (Vazyme, Q312–02). qPCR reactions using primers targeting ACAT1 (forward 5′-GGAGGTGAAGGACAAGCTCC-3′ and reverse 5′-TCTACAGCAGCGTCAGCAAA-3′), ECHS1(forward 5′- TCCTGACTGGAGCACCTTCT3′ and reverse 5′-GCATCTGTATGAAGGCAGCA3′) and ACADM (forward 5′- ACAACGTGAACCAGGATTAG-3′ and reverse 5′-TGGCAAATTTACGAGCAGTA-3′), with GAPDH (forward 5′-GCACCACCAACTGCTTA-3′ and reverse 5′-AGTAGAGGCAGGGATGAT-3′) as the loading control.

### 2.8 Univariate Cox analysis, multivariate Cox analysis, and LMRS

The survival v3.3.1 package in R was utilized for conducting kaplan-meier analysis, and constructing univariate Cox regression models (Cox models) and multivariate Cox models. Based on the prognostic biomarkers we found, we constructed a lipid metabolic risk score (LMRS):


$$\text{LMRS}=\sum_{\text{i}}\;{\text{β}}_{\text{i}}\times {\text{EXP}}_{\text{i}}$$

β_i_ is the regression coefficient of gene i in the univariate Cox regression model and is used as the weight of gene i in the calculation formula. EXP_i_ indicates the expression level of gene i.

Univariate Cox models were built based on survival time, survival outcome, gene expression levels or the LMRS. Multivariate Cox models were constructed based on survival time, survival outcome, gene expression levels or LMRS, along with age and gender. For Cox models, a model was considered reliable when the p-value from the model’s wald test was less than 0.05. Factors with wald test p-values less than 0.05 were considered to significantly influence survival in a reliable Cox model. Kaplan-meier analysis was employed to compare the survival rates of different sample groups and to plot survival curves. The log-rank test was utilized as a non-parametric method for comparing survival rates, with a p-value less than 0.05 indicating significant differences in survival rates between two groups. Additionally, the time-dependent prognostic significance of LMRS was investigated using the timeROC v0.4.0 package in R.

### 2.9 Machine learning model

We used the glmnet v4.1 package in R to build a lasso classification model using expression data from all ccRCC samples obtained from the TCGA-KIRC cohort, aiming to select important variables significantly associated with patient prognosis. Variables with non-zero coefficients in the lasso model are considered important for model.

In addition to this, the samples from the TCGA-KIRC cohort were divided into a training set and an internal validation set in a 7:3 ratio. We used the caret v6.0.92 package in R to build machine learning models. To construct the best-performing models, we included ten of the most common machine learning algorithms: bayesian generalized linear model (bayesglm), random forest (rf), neural network (nnet), k-nearest neighbors (knn), support vector machines with linear kernel (svmLinear), support vector machines with radial basis function kernel (svmRadial), classification and regression tree (rpart), linear discriminant analysis (lda), linear discriminant analysis with stepwise feature selection (stepLDA), and bagged AdaBoost (AdaBag). The caret package automatically selects the optimal values for each important parameter affecting model performance during training, thus constructing the best-performing machine learning models. Subsequently, we validated the ten trained models using the internal validation set. By plotting the receiver operating characteristic (ROC) curves, we selected the model with the highest area under curve (AUC) value as the final best machine learning model. We then applied the best model to the external validation set, and the AUC value reflected the predictive performance of the model.

### 2.10 Evaluation of immune cell infiltration levels, ESTIMATE evaluation, and TIDE score

We used the immunedeconv v2.0.3 (32) package in R to evaluate the immune cell infiltration levels in the TCGA-KIRC cohort. Immunedeconv provides a unified interface for multiple immune cell infiltration algorithms, such as quantiseq and cibersort, and accepts the TPM matrix as input for evaluation. We used the quantiseq (33) algorithm to assess the relative infiltration levels of 11 cell types in the TME of each sample. Additionally, we employed the cibersort (34) algorithm to assess the relative infiltration levels of 22 cell types in the TME of each sample. Furthermore, the estimate v1.0.13 (35) package in R was used to evaluate the StromalScore, ImmuneScore, and ESTIMATEScore of the TME for each sample in the TCGA-KIRC cohort. The tumor immune dysfunction and exclusion (TIDE) scores for each sample in the TCGA-KIRC cohort were obtained from TIDE (http://tide.dfci.harvard.edu/, accessed on 17 October 2023) (36).

### 2.11 Somatic mutation analysis

Previously published work provided a high-quality assessment of the somatic mutation landscape in the TCGA cohort and made the results available for download in the form of MAF files (accessed on 23 June 2023) (37). We used the maftools v2.12.0 package in R to read the downloaded MAF files. Oncoplot was used to display the mutation frequencies of genes. Additionally, we evaluated the tumor mutation burden (TMB) for each sample.

### 2.12 Drug sensitivity analysis

Transcriptomic alterations in cancer patients strongly influence their response to anticancer drugs (38). The development of the oncoPredict v0.1.0 (39) package in R helped us predict patient responses to anticancer drugs based on the bulk transcriptomic data. Using gene expression matrices and drug response matrices from GDSC (accessed on 28 May 2023) (40) as references, we evaluated the drug sensitivity (IC50 values) of the TCGA-KIRC cohort’s ccRCC samples to 449 drugs. The assessed drug sensitivity values represented the IC50 values of the samples’ response to the drugs. A smaller IC50 value indicates a stronger response to the drug. Additionally, we obtained detailed information on the drug targets from GDSC.

### 2.13 Statistical analysis

All statistical analyses were conducted using R v4.2.2. The psych v2.2.5 package was used for correlation analysis. Unless otherwise specified, all correlation analyses were pearson correlations. The stats v4.2.2 package was used for related statistical tests, such as the T-test. Plotting was primarily done using packages such as pheatmap v1.0.12, ggplot2 v 3.3.6, and ggpubr v 0.4.0 in R.

## 3 Results

### 3.1 Identification of lipid metabolism dysregulation genes associated with prognosis through bulk transcriptome analysis

**Figure 1:**
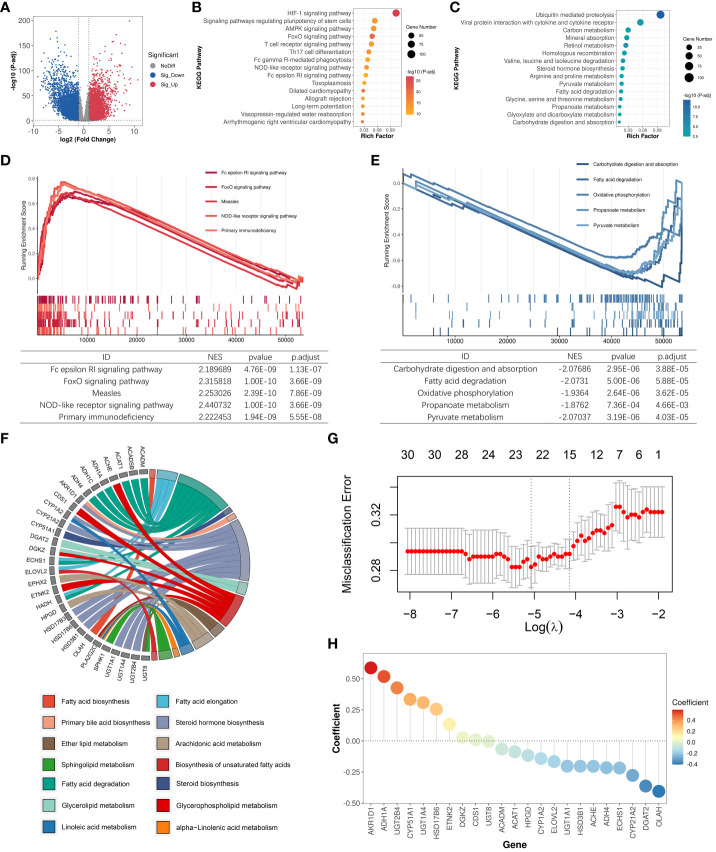
Identification of LMDPs through bulk transcriptome analysis. **(A)** Differential expression gene (DEG) analysis between ccRCC samples and normal tissues. Red indicates significantly upregulated DEGs in ccRCC samples. Blue indicates significantly downregulated DEGs in ccRCC samples. Gray indicates no significant difference in gene expression between the two groups. **(B)** KEGG functional enrichment analysis (top 15) of significantly upregulated DEGs in ccRCC samples. **(C)** KEGG functional enrichment analysis (top 15) of significantly downregulated DEGs in ccRCC samples. **(D)** GSEA analysis shows the top 5 KEGG pathways significantly associated with the ccRCC phenotype. **(E)** GSEA analysis shows the top 5 KEGG pathways significantly associated with normal tissue phenotype. **(F)** Functional annotation of 30 LMDPs. **(G)** Misclassification error corresponding to different lambda parameters in the lasso model. **(H)** Coefficients corresponding to each gene in the lasso model.

In our study, we incorporated 14 lipid metabolism-related pathways from KEGG, encompassing a total of 372 lipid metabolism-related genes (**Supplementary Table S1**). To investigate the dysregulation of lipid metabolism-related genes in clear cell renal cell carcinoma (ccRCC), we collected bulk transcriptome data from 602 samples within the TCGA-KIRC cohort (531 tumor samples; 71 normal samples) and conducted a comprehensive analysis. The analysis of differentially expressed genes (DEGs) between tumor and normal samples revealed significant upregulation of expression in 6336 genes and significant downregulation in 5293 genes within the ccRCC samples (**Figure 1A**). Functional analysis of the DEGs indicated that the upregulated genes in ccRCC were primarily enriched in pathways such as HIF-1 signaling pathway and FoxO signaling pathway, whereas downregulated genes were predominantly enriched in pathways including retinol metabolism, carbon metabolism, and fatty acid degradation (**Figures 1B, C**). Gene set enrichment analysis (GSEA) corroborated these findings, revealing significant upregulation of pathways such as the NOD-like receptor signaling pathway and FoxO signaling pathway in ccRCC (**Figure 1D**). Conversely, pathways like carbohydrate digestion and absorption, fatty acid degradation, and pyruvate metabolism were significantly associated with the phenotypes of normal samples and downregulated in ccRCC samples (**Figure 1E**). Moreover, we identified a total of 134 dysregulated lipid metabolism genes, of which 50 genes exhibited significantly increased expression and 84 genes displayed significant downregulation in ccRCC (**Supplementary Table S2**).

We constructed univariate and multivariate Cox regression models to explore the association between dysregulated lipid metabolism genes and the prognosis of ccRCC patients (**Supplementary Table S3**). Under the condition that the significance level of the univariate Cox model and multivariate Cox model were met at the same time, we discovered that the expression of 30 genes among 134 dysregulated lipid metabolism genes identified by differential expression analysis was significantly associated with the prognosis of ccRCC patients. Among the 30 lipid metabolism dysregulation genes associated with prognosis (LMDPs), 9 genes were implicated in the steroid hormone biosynthesis pathway, followed by 8 genes in the fatty acid degradation pathway (**Figure 1F**). We further constructed a lasso classification machine learning model to select important variables associated with prognosis (**Supplementary Table S4**). Under the optimal lambda parameter, a total of 23 significant LMDPs were identified (**Figures 1G, H**). These LMDPs serve as our primary focus for subsequent analysis.

### 3.2 Further integrates single-cell transcriptomic analysis to identify key prognostic signatures

**Figure 2:**
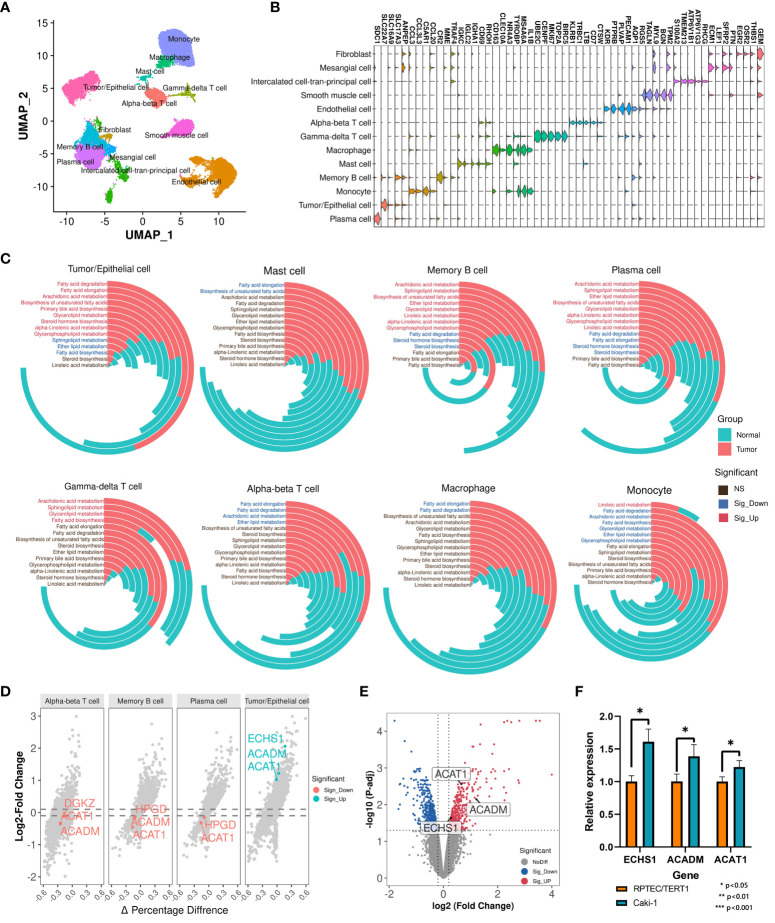
Single-cell transcriptomic analysis identifies key prognostic signatures. **(A)** Cell annotation of 25,558 cells. **(B)** Display of the marker genes for each cell subtype. **(C)** Comparison of activation levels of lipid metabolism pathways in each cell subtype between tumor samples and normal samples. The height of the histogram represents the mean level of pathway activation. **(D)** Differential expression landscape of key LMDPs in each cell subtype. **(E)** DEG analysis between Caki-1 and RPTEC/TERT1. Red indicates significantly upregulated DEGs in Caki-1. Blue indicates significantly downregulated DEGs in Caki-1. **(F)** qRT-PCR results of genes (ACAT1, ACADM, and ECHS1) in Caki-1 and RPTEC/TERT1 cell lines. '*' indicates that the p-value from the t-test is less than 0.05.

Bulk RNA-Seq reflects a complex transcriptional landscape of all cell populations within the tumor microenvironment (TME). To finely explore the landscape of lipid metabolism reprogramming in various cells within the tumor microenvironment (TME) of ccRCC patients, we conducted a study on single-cell transcriptomes of 7 ccRCC samples and 6 normal samples from the GSE159115 dataset. After quality control, 25,558 high-quality cells were retained and clustered into 13 major cell types: Tumor/Epithelial cell, Plasma cell, Monocyte, Memory B cell, Mast cell, Macrophage, Gamma-delta T cell, Alpha-beta T cell, Endothelial cell, Smooth muscle cell, Intercalated cell-tran-principal cell, Mesangial cell, Fibroblast (**Figures 2A, B**). High lipid absorption, storage, and fat generation occur in various cancers, contributing to the rapid growth of tumors (41). We evaluated the activation levels of 14 lipid metabolism-related pathways in various cell subtypes. Subsequently, we used the U-test to compare the differences in the activation levels of metabolic pathways between tumor samples and normal samples (**Supplementary Table S5** **;** **Figure 2C**). For Tumor/Epithelial cells, we found that the activation levels of 9 lipid metabolism pathways were significantly higher in tumor samples than in normal samples. In addition, lipid metabolism undergoes varying degrees of reprogramming in seven other immune cell subtypes among cancer patients. Thus, within the nutritionally deficient tumor microenvironment, as nutrient levels fluctuate, tumor cells utilize lipid metabolism reprogramming to enhance their survival environment, leading to metabolic competition that may affect the normal function of immune cells.

Based on single-cell transcriptome data, we analyzed the transcriptional variation landscape of various cell subtypes in cancer samples compared to normal samples (**Supplementary Table S6**). We then overlapped all identified differentially expressed genes in each cell subtype with the 23 LMDPs identified through bulk transcriptome analysis, revealing 5 overlaps. These included 4 protective genes (ACADM, ACAT1, ECHS1, HPGD) and 1 risk gene (DGKZ) (**Figure 2D**, **Supplementary Figure S1A**). The expression levels of three protective genes (ACAT1, ACADM, and ECHS1) were significantly higher in Tumor/Epithelial cells of tumor patients than in normal samples at single-cell level. To verify this result, we collected microarray data from the ccRCC cell line (Caki-1) and the human renal epithelial cell line (RPTEC/TERT1), and found that the expression levels of three genes (ACAT1, ACADM, and ECHS1) were significantly higher in Caki-1 (**Supplementary Table S7** **;** **Figure 2E** **).** Next, we also found that the expression levels of three genes (ACAT1, ACADM, and ECHS1) were significantly higher in Caki-1 cell line than RPTEC/TERT1 through qRT-PCR experiments (**Figure 2F**). This was consistent with our findings at the single-cell level. Conversely, in adaptive immune cells of tumor samples (Alpha-beta T cell, Plasma cell, Memory B cell), the expression level of ACAT1 was significantly lower than in normal samples. Bulk transcriptome analysis based on the TME indicated that relative to normal samples, the expression levels of the 4 protective genes were significantly downregulated in ccRCC samples, while the expression level of the risk gene DGKZ was significantly upregulated (**Supplementary Figure S1B**), consistent with the differentially expressed genes results based on ANOVA analysis from GEPIA2 (**Supplementary Figure S1C**). In the breakdown of fatty acids via the beta-oxidation pathway into acetyl-CoA, the expression product of ACAT1 catalyzes the final step of this reaction. ACAT1, ACADM, and ECHS1 are all involved in the fatty acid degradation pathway. ACADM and ECHS1 also play critical roles in this beta-oxidation process. The expression product of HPGD catalyzes the dehydrogenation reaction of a series of hydroxylated polyunsaturated fatty acids, participating in the arachidonic acid metabolism pathway. The risk gene DGKZ is a diacylglycerol kinase involved in the glycerolipid metabolism and glycerophospholipid metabolism metabolic pathways. In summary, these 5 key LMDPs identified through bulk and single-cell transcriptome analysis may play important roles in lipid metabolism reprogramming within the TME and could serve as prognostic signatures for ccRCC.

### 3.3 Construction and validation of cancer occurrence and prognosis models based on key prognostic signatures

**Figure 3:**
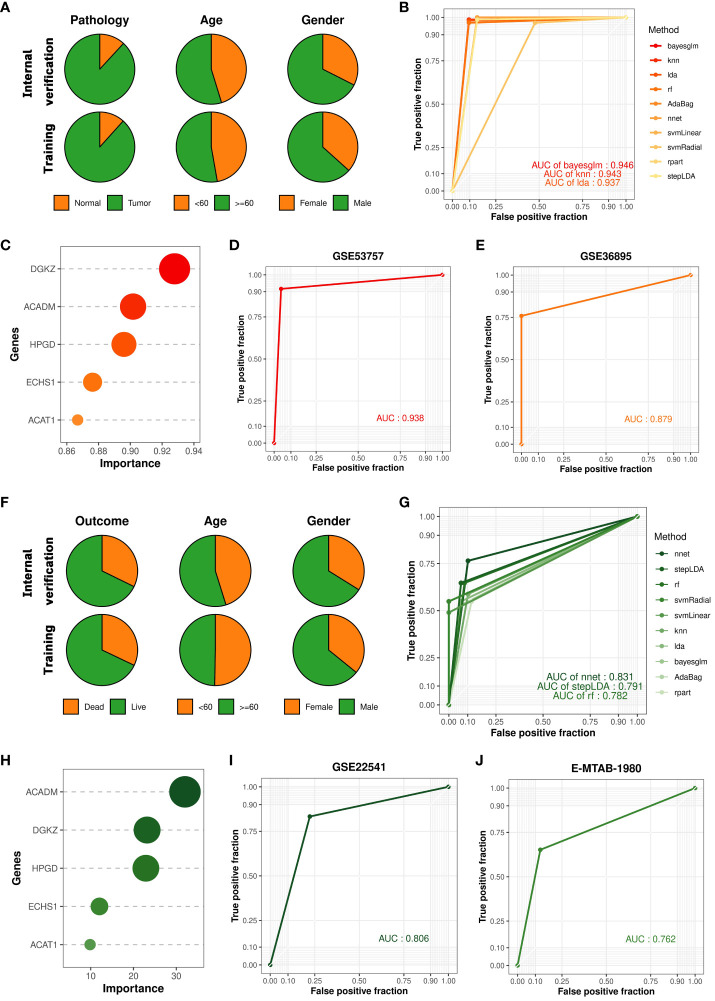
Construction and validation of cancer occurrence and prognosis models based on key prognostic signatures. **(A)** Clinical features of samples in the internal validation set and training set used to construct the cancer occurrence model. **(B)** ROC curves of 10 cancer occurrence models applied to the internal validation set. **(C)** Importance of each factor in the bayesglm model. **(D)** ROC curve of the bayesglm model applied to the GSE53757 dataset. **(E)** ROC curve of the bayesglm model applied to the GSE36895 dataset. **(F)** Clinical features of samples in the internal validation set and training set used to construct the cancer prognosis model. **(G)** ROC curves of 10 cancer prognosis models applied to the internal validation set. **(H)** Importance of each factor in the nnet model. **(I)** ROC curve of the nnet model applied to the GSE22541 dataset. **(J)** ROC curve of the nnet model applied to the E-MTAB-1980 dataset.

Renal cell carcinoma has been described as a metabolic disease, with alterations in metabolism leading to diverse cancer etiologies, particularly changes in lipid metabolism (42). Therefore, lipid metabolism may hold potential value in the diagnosis of ccRCC occurrence and progression. To explore the applicability of lipid metabolism-related genes in predicting the occurrence and prognosis of ccRCC, we constructed machine learning models to assess their diagnostic performance. Leveraging the 5 key prognostic signatures identified through combined bulk and single-cell transcriptome analysis, we initially developed a machine learning model for predicting the occurrence of ccRCC, distinguishing ccRCC samples and normal samples. We partitioned the entire TCGA-KIRC cohort into a training set and an internal validation set in a 7:3 ratio, and found no significant differences in the distribution of various clinical features between the two datasets (**Figure 3A**). To build the optimal machine learning model, we included 10 of the most common machine learning algorithms. Under the optimal parameters for each model, the bayesian generalized linear model (bayesglm) exhibited the best performance, with an AUC value of 0.946 for predictions on the internal validation set (**Figure 3B**). For the bayesglm model, the risk gene DGKZ is the most important factor (**Figure 3C**). Furthermore, to further evaluate the performance of the model we constructed, we included two external validation datasets, GSE53757 (72 ccRCC tumor samples; 72 normal kidney samples) and GSE36895 (29 ccRCC tumor samples; 23 normal kidney cortex samples). In both datasets, our bayesglm model effectively distinguished ccRCC samples and normal samples, with AUC values of 0.938 (**Figures 3D**) and 0.879 (**Figures 3E**), respectively, indicating the robustness and accuracy of the model.

The results of survival analysis from HPA (www.proteinatlas.org/) confirmed the association between the expression of five key prognostic signatures and the survival of ccRCC patients, which was consistent with our findings (**Supplementary Figure S2**). To evaluate the potential of prognostic signatures we identified in predicting ccRCC patient prognosis, we employed a similar approach to construct a machine learning model for predicting the survival outcomes (death or survival) of ccRCC patients. We retained the cancer samples from the TCGA-KIRC cohort and split the dataset into a training set and an internal validation set in a 7:3 ratio, with no significant differences in the distribution of various clinical features between the two datasets (**Figure 3F**). Under the optimal parameters for each of the 10 machine learning algorithms, the neural network (nnet) model exhibited the best performance, with an AUC value of 0.831 for predictions on the internal validation set (**Figure 3G**). Regarding the constructed nnet model, the protective gene ACADM demonstrated the highest importance (**Figure 3H**). To validate the model’s performance, we included two external validation datasets, GSE22541 (6 dead samples; 18 alive samples) and E-MTAB-1980 (23 dead samples; 78 alive samples). Both datasets investigated the transcriptomes of multiple ccRCC patients and provided clear survival outcome information for each sample. Based on the transcriptome data from the two cohorts, we utilized the constructed nnet model to predict the survival outcomes of each sample. We found that the nnet model we developed performed well in both datasets, with AUC values of 0.806 (**Figures 3I**) and 0.762 (**Figures 3J**), respectively. These findings suggested that prognostic signatures we found had great application value in indicating the occurrence of ccRCC and patient prognosis.

### 3.4 Construction of LMRS and its association with genomic characteristics

**Figure 4:**
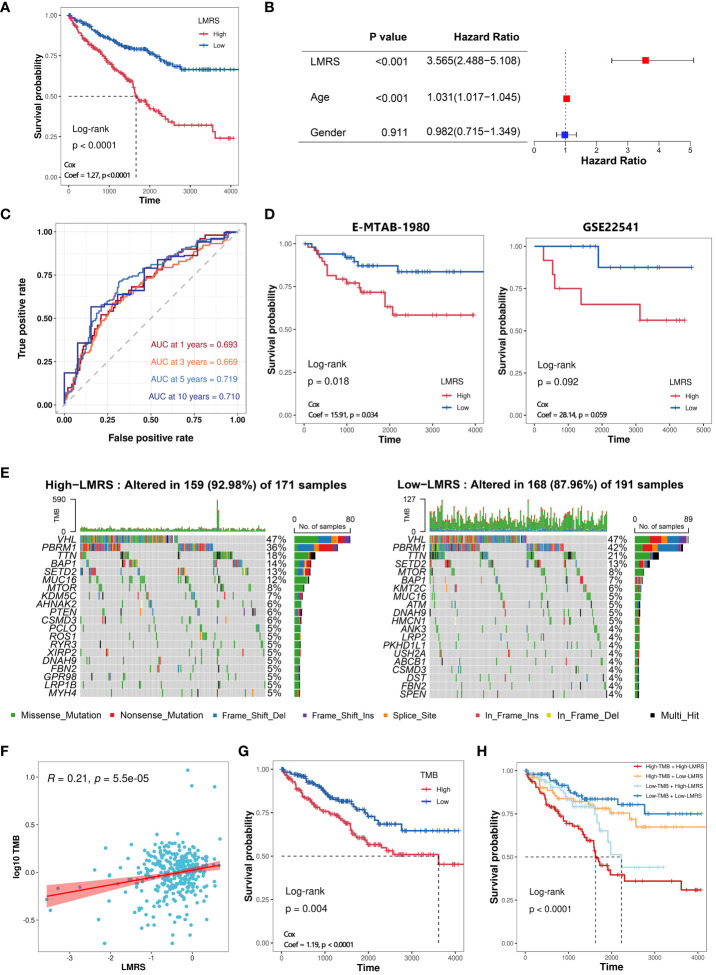
Construction of LMRS and its association with genomic characteristics. **(A)** Kaplan-meier analysis of High-LMRS and Low-LMRS groups in TCGA-KIRC cohort. **(B)** Multivariate Cox model constructed with LMRS, age, and gender. **(C)** Time-dependent ROC curve of LMRS. **(D)** Kaplan-meier analysis of High-LMRS and Low-LMRS groups in E-MTAB-1980 and GSE22541 cohort. **(E)** Top 20 genes with the highest mutation frequency in different LMRS groups. **(F)** Pearson correlation analysis between LMRS and TMB. **(G)** Kaplan-meier analysis of different TMB groups. **(H)** Kaplan-meier analysis combining LMRS and TMB.

To further explore the clinical application value of lipid metabolism-related prognostic signature, we constructed the lipid metabolism risk score (LMRS) as a risk index in ccRCC. LMRS is a simple linear model based on the gene expression levels of key prognostic signatures and their univariate Cox regression coefficients. The calculation formula for LMRS is as follows: Risk score = 0.0272 * Exp (DGKZ) + (−0.0059) * Exp (ACADM) + (−0.0033) * Exp (ACAT1) + (−0.0012) * Exp (ECHS1) + (−0.0186) * Exp (HPGD). Using the median LMRS of the patients, the ccRCC samples from the TCGA-KIRC cohort were divided into two groups: High-LMRS and Low-LMRS (**Supplementary Table S8**). Kaplan-meier analysis revealed a significantly poorer prognosis in the High-LMRS group (log-rank test, p value< 0.0001) (**Figure 4A**). Subsequently, we constructed a univariate Cox regression model and found that LMRS was significantly associated with poor prognosis in the KIRC samples (HR = 3.56, p value< 0.0001). In the multivariate Cox regression model considering the LMRS, patient age, and patient gender, the relationship between LMRS and poor prognosis remained significant (**Figure 4B**). Furthermore, there were no significant differences in LMRS among different age and gender groups (**Supplementary Figures S3A, B**). These findings suggest that LMRS is an independent prognostic factor and is significantly associated with poor prognosis in ccRCC patients. Time-dependent ROC curve analysis revealed that the LMRS predicted the one-year, three-year, five-year, and ten-year survival outcomes of ccRCC patients with AUC values of 0.693, 0.669, 0.719, and 0.710, respectively (**Figure 4C**). This indicates that LMRS has good sensitivity and specificity for predicting patient prognosis. Then, we also observed that the high LMRS group had a lower survival probability in the E-MTAB-1980 (log-rank test, p value = 0.018) and GSE22541 cohorts (log-rank test, p value = 0.092) (**Supplementary Table S9** **;** **Figure 4D**).

Genomic variations are not only the driving forces behind the development of tumors but also major characteristics of cancer (43). The proportion of mutation events was found to be higher in the High-LMRS group (92.98%) compared to the Low-LMRS group (87.96%) (**Figure 4E**). The most common mutations in both the High-LMRS and Low-LMRS groups were VHL, PBRM1, and TTN. The expression product of the von Hippel-Lindau tumor suppressor (VHL) plays a critical role in cellular oxygen sensing by targeting hypoxia-inducible factors for ubiquitination and proteasomal degradation, and it has emerged as a potential therapeutic target for the treatment of late-stage ccRCC (44). Polybromo 1 (PBRM1) encodes a subunit of the ATP-dependent chromatin remodeling complex, and its mutations are associated with primary ccRCC (45, 46). Titin (TTN) encodes a highly abundant sarcomeric protein, and its mutations are implicated in the development of various cancers (47–49). Tumor mutational burden (TMB) refers to the number of variant bases per million bases in tumor tissue and is an emerging biomarker increasingly used for predicting patient prognosis. We found a significant positive correlation between TMB and LMRS (Coef = 0.21, p value< 0.001) (**Supplementary Table S10**; **Figure 4F**). Kaplan-meier analysis revealed that ccRCC patients in the High-TMB group had a poorer prognosis (log-rank test, p value< 0.0001) (**Figure 4G**). Furthermore, when combining TMB and LMRS, a better prediction of prognosis was achieved, as the ccRCC samples in the High-TMB with High-LMRS group exhibited significantly poorer prognosis (log-rank test, p value< 0.0001) (**Figure 4H**).

### 3.5 Immune landscape in the tumor microenvironment of different LMRS groups

**Figure 5:**
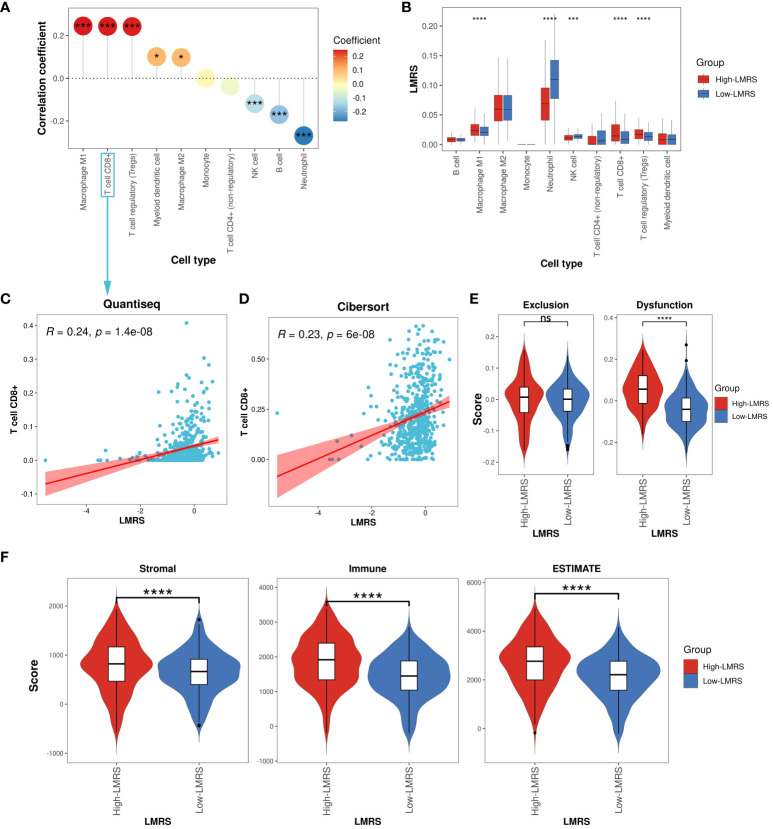
Immune landscape of different LMRS groups. **(A)** Correlation analysis between LMRS and immune cell infiltration levels. **(B)** Comparison of immune cell infiltration levels between different LMRS groups. **(C)** Correlation analysis between LMRS and T cell CD8+ infiltration levels evaluated by the quantiseq. **(D)** Correlation analysis between LMRS and T cell CD8+ infiltration levels evaluated by the cibersort algorithm. **(E)** Comparison of exclusion score and dysfunction score between different LMRS groups (ns, non-significant; ****, p value< 0.0001). **(F)** Comparison of stromal score, immune score, and estimate score between different LMRS groups (****, p value< 0.0001). '*' indicates that the p-value is less than 0.05, and '***' indicates that the p-value is less than 0.001.

Different types of infiltrating immune cells in the tumor microenvironment (TME) have varying impacts on tumor progression, which may differ depending on the cancer type (50). Clinical studies on immune cells infiltrating the TME have confirmed the roles of cytotoxic T cells and tumor-associated macrophages in cancer progression (51, 52). To further investigate the immune characteristics of different LMRS groups, we employed the quantiseq algorithm to evaluate the relative infiltration levels of 11 immune cell types (B cell, macrophage M1, macrophage M2, monocyte, neutrophil, natural killer (NK) cell, T cell CD4+ (non-regulatory), T cell CD8+, T cell regulatory (Tregs), myeloid dendritic cell, uncharacterized cell) in the TCGA-KIRC cohort of ccRCC samples (**Supplementary Table S11**). The uncharacterized cell was not included in our subsequent analysis. We observed a significant positive correlation between LMRS and the infiltration levels of macrophage M1, T cell CD8+, T cell regulatory (Tregs), myeloid dendritic cell, and macrophage M2 (**Figure 5A**), while a significant negative correlation was found with the infiltration levels of NK cells, B cells, and neutrophils. Furthermore, compared to the Low-LMRS group, the High-LMRS group exhibited significantly higher infiltration levels of macrophage M1, T cell CD8+, and T cell regulatory (Tregs), and significantly lower infiltration levels of neutrophils and NK cells (**Figure 5B**).

In numerous human malignancies, the presence of T cells has been associated with improved patient prognosis (53). However, we found a significant positive correlation between LMRS and the infiltration levels of T cell CD8+ (Coef = 0.24, p value< 0.001) (**Figure 5C**), with the High-LMRS group exhibiting a poorer prognosis. To elucidate this phenomenon, we re-evaluated the immune infiltration levels of the TCGA-KIRC cohort of ccRCC samples using the cibersort algorithm and similarly observed a significant positive correlation between LMRS and T cell CD8+ infiltration levels (Coef = 0.23, p value< 0.001) (**Figure 5D**). Indeed, research evidence suggests elevated levels of T cells CD8+ in late-stage ccRCC, impacting patient response to immunotherapy (54). The TIDE algorithm evaluated the tumor-infiltrating cytotoxic T cell exclusion score and dysfunction score. We found that the High-LMRS group exhibited significantly higher T cell dysfunction scores (T test, p value< 0.0001). Although the T cell exclusion score was higher in the High-LMRS group, there was no statistically significant difference between the two groups (**Figure 5E**). Additionally, studies have indicated a link between lipid accumulation and T cell dysfunction (55). Therefore, we speculate that the tumor-infiltrating T cell CD8+ in ccRCC samples may primarily be in an immunosuppressive state, unable to exert normal cytotoxic functions, thus affecting patient prognosis. Furthermore, we utilized the ESTIMATE algorithm to assess the stromal score, immune score, and overall ESTIMATE score of the TCGA-KIRC cohort of ccRCC samples, finding all three scores significantly higher in the High-LMRS group (**Figure 5F**) (T test, p value< 0.0001). Overall, the High-LMRS group exhibited higher levels of immune cell infiltration in the TME, potentially benefiting more from immunotherapy.

### 3.6 Higher LMRS associated with enhanced responsiveness to immunotherapy

**Figure 6:**
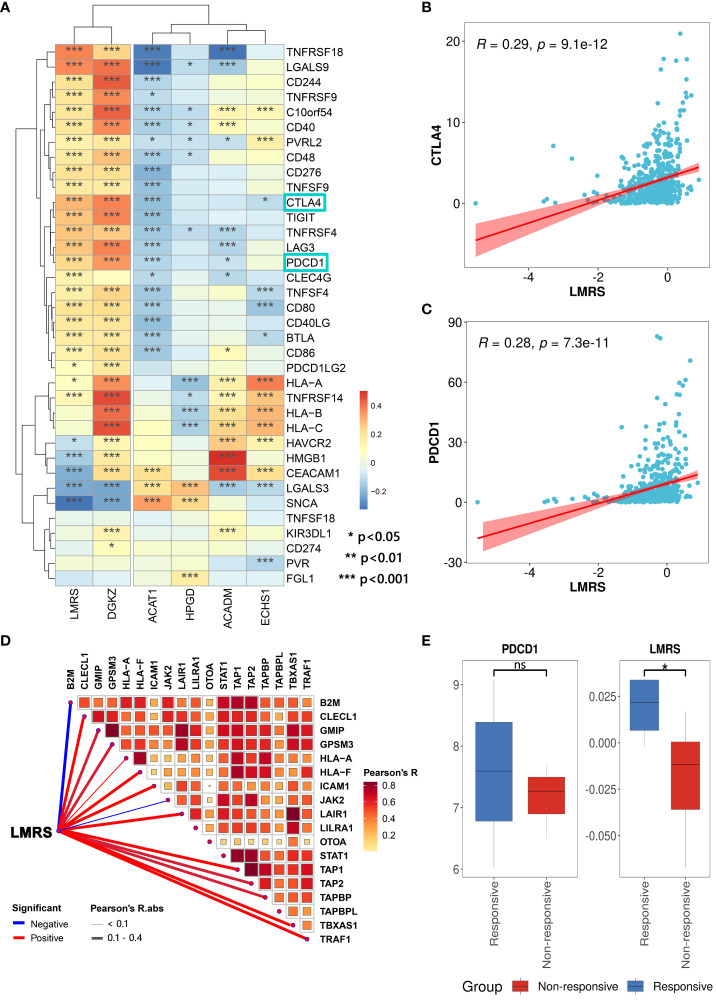
Association of LMRS with immunotherapy efficacy. **(A)** Correlation between prognostic signatures, LMRS, and immune checkpoint genes (*, p value< 0.05; **, p value< 0.01; ***, p value< 0.001). **(B)** Correlation analysis between LMRS and CTLA4. **(C)** Correlation analysis between LMRS and PDCD1. **(D)** Pearson correlation analysis results between LMRS and essential genes for immunotherapy. **(E)** Comparison of the expression levels of PDCD1 and LMRS between different response groups to immunotherapy (ns, non-significant; *, p< 0.05).

Therapeutic interventions targeting lipid metabolism can restrain tumor cell growth, and alleviate immune suppression within the tumor microenvironment, thereby enhancing responsiveness to immune checkpoint blockade therapy (56). To evaluate the predictive potential of LMRS for the efficacy of immune checkpoint blockade therapy, we collected 36 widely utilized immune checkpoint genes (57). We found a widespread and significant positive correlation between LMRS and immune checkpoint genes in the TCGA-KIRC cohort (**Figure 6A**). Additionally, in the analysis of the correlation with immune checkpoint genes, LMRS exhibited a similar landscape to the risk gene DGKZ. Moreover, PDCD1 (PD1), as an immune inhibitory receptor, is expressed in various types of immune system cells, particularly cytotoxic T cells (58). The expression of PDCD1 and CTLA4 is commonly associated with improved immunotherapeutic efficacy (59, 60). We observed a significant positive correlation between the two classical immune checkpoints (PDCD1 and CTLA4) with LMRS (**Figures 6B, C**). These findings suggest that higher LMRS might be associated with improved responsiveness to immune checkpoint blockade therapy.

During the course of patient response to immune checkpoint blockade therapy, the support of products of certain essential genes is required. These essential genes play a crucial role in antigen presentation and the cytotoxic capability of T cell CD8+, directly impacting the efficacy of immune checkpoint blockade therapy (61). We compiled 18 essential genes for immune checkpoint blockade therapy (62) and found a significant positive correlation between LMRS and the expression of multiple essential genes (**Figure 6D**). Subsequently, we incorporated a cohort of immune therapy (GSE67501), which studied the transcriptional landscape of 11 ccRCC patients before receiving nivolumab (anti-PDCD1) treatment and carefully documented the response of each patient to immunotherapy after nivolumab treatment (7 non-responsive samples and 4 responsive samples) (**Supplementary Table S12**). We found that the expression of PDCD1 in the responsive group was higher than in the non-responsive group, although not statistically significant (**Figure 6E**). Meanwhile, the LMRS of the responsive group was significantly higher than the non-responsive group (T test, p value = 0.030). In conclusion, LMRS can be used to predict the response of ccRCC patients to immune checkpoint blockade therapy. Patients with higher LMRS are more likely to benefit from immunotherapy.

### 3.7 Significance of LMRS in guiding anticancer drug selection

**Figure 7:**
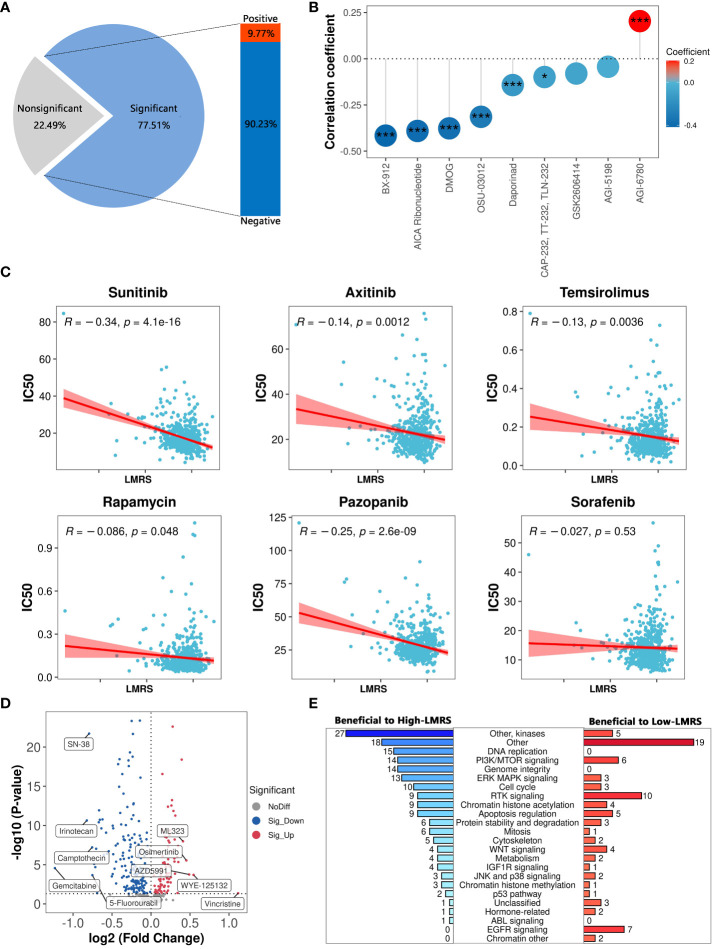
The guiding significance of LMRS in the selection of anticancer drugs. **(A)** Statistics of correlation analysis between IC50 value of anticancer drugs and LMRS. **(B)** Correlation between LMRS and 9 targeted metabolic pathway anticancer drugs (***, p value< 0.001; *, p value< 0.05). **(C)** Correlation analysis between LMRS and 6 commonly used anticancer drugs. **(D)** Differential analysis of IC50 values of anticancer drugs between different LMRS groups. Blue represents drugs with lower IC50 values in the High-LMRS group. Red represents drugs with higher IC50 values in the High-LMRS group. The top 10 drugs with the largest differences in IC50 values between the two groups are specifically marked. **(E)** Statistical analysis of the targets of drugs with significant differences in IC50 values between the two groups.

Current treatments for ccRCC often involve cytokine therapy and targeted drug therapy, yet they fail to generate a universal and enduring complete remission response (63). To explore the guidance significance of LMRS in anticancer drug therapy, we employed oncoPredict to predict the drug sensitivity (IC50 values) of 449 drugs included in GDSC for ccRCC samples in the TCGA-KIRC cohort (**Supplementary Tables S13**, **S14**). Lower IC50 values indicate higher sensitivity of the samples to anticancer drugs. Our study identified a significant correlation between LMRS and IC50 values of 348 drugs, with 90.23% showing a significant negative correlation (**Supplementary Table S15** **;** **Figure 7A**). Considering the target information of the drugs, we identified 9 drugs targeting metabolic pathways. Among them, LMRS exhibited a significant negative correlation with the IC50 values of 6 drugs targeting metabolic pathways (BX-912, AICA ribonucleotide, DMOG, OSU-03012, daporinad, CAP-232), and only a significant positive correlation with AGI-6780 (**Figure 7B**).

Sunitinib, an orally active small molecule tyrosine kinase inhibitor, with potent anti-angiogenic and antitumor activities, is widely used as a first-line treatment for renal cell carcinoma (64). Additionally, other drugs such as axitinib are also commonly used in the first-line treatment of ccRCC patients (6, 7). We found a significant negative correlation between LMRS and the IC50 values of sunitinib, axitinib, temsirolimus, rapamycin, and pazopanib (**Figure 7C**). While LMRS exhibited a negative correlation with the IC50 value of sorafenib, it was not statistically significant.

The structural heterogeneity of the tumor microenvironment in ccRCC patients leads to different clinical outcomes and postoperative recurrence risks, thus emphasizing the urgent need for personalized treatment strategies (65, 66). We further compared the drug sensitivity between the High-LMRS and Low-LMRS groups, discovering that the High-LMRS group showed greater sensitivity to 178 drugs such as gemcitabine (pyrimidine antimetabolite targeting DNA replication) and irinotecan (topoisomerase I inhibitor targeting DNA topology) (**Figure 7D**). In contrast, the Low-LMRS group demonstrated increased sensitivity to 86 drugs, including vincristine (microtubule assembly inhibitor) and WYE-125132 (mTOR inhibitor). Statistical analysis of the targeted pathways for each drug revealed that the sensitive drugs in the High-LMRS group primarily targeted kinases and DNA replication (**Figure 7E**). Conversely, the sensitive drugs in the Low-LMRS group mainly targeted RTK signaling, EGFR signaling, and PI3K/MTOR signaling pathways. In summary, these findings indicate that the LMRS we constructed could offer guidance for targeted therapy, facilitating the development of personalized treatment regimens for ccRCC.

## 4 Discussion

Renal cell carcinoma (RCC) refers to cancer originating in the renal epithelium, and clear cell renal cell carcinoma (ccRCC) is the most common subtype of RCC (67). Histologically, ccRCC is defined by malignant epithelial cells with clear cytoplasm, attributed to the accumulation of abundant lipid droplets within the cytoplasm (68). While the 5-year survival rate for stage I ccRCC patients approaches 90%, it drops below 10% for patients with stage IV disease (69). Metabolic dysregulation is a recognized hallmark of cancer and an enticing target for cancer therapy (70). Elevated lipid levels are associated with tumor progression and pathophysiology of ccRCC (12). Additionally, aberrant lipid metabolic activities within the tumor microenvironment (TME) promote tumor growth and suppress the activation of immune cells, thereby attenuating anti-tumor immunity (71). And ccRCC often exhibits significant tumor heterogeneity, presenting a major challenge for anticancer therapies (72, 73). Although there is a strong correlation between the pathological stage and the risk of death in ccRCC, relying solely on the pathological stage is insufficient to inform the prognosis of most patients (74). Therefore, it is essential to comprehensively investigate lipid metabolism-related genes as potential novel prognostic signatures for ccRCC and construct corresponding risk indices to predict the prognosis and treatment response of ccRCC patients.

A previous study developed a risk model consisting of four genes (ACADM, ACAT1, CPT1B, and HACD1) for assessing the risk of ccRCC. This study provides a valuable research paradigm and has some findings similar to ours. However, it did not focus on the association between lipid metabolism and ccRCC patients at the single-cell level (75). In our study, we identified five lipid metabolism-related prognostic signatures, including four protective genes (ACADM, ACAT1, ECHS1, HPGD) and one risk gene (DGKZ), through the integration of bulk and single-cell transcriptome analyses. Bulk transcriptome analysis revealed the downregulation of the four protective genes and upregulation of the risk gene in ccRCC samples relative to normal kidney tissue samples. The analysis of single-cell transcriptomes shows that these five genes exhibit significant differences in expression levels across multiple cell types in tumor samples compared to normal samples. ACAT1, ACADM, and ECHS1 are all involved in the fatty acid degradation pathway. ACAT1, serving as acetyl-CoA acetyltransferase, catalyzes the final step of the reaction where fatty acids are broken down into acetyl-CoA via the beta-oxidation pathway. Furthermore, the expression product of ACADM (medium-chain specific acyl-CoA dehydrogenase) is one of the key enzymes catalyzing the first step of this beta-oxidation reaction, while ECHS1 (enoyl-CoA hydratase, short chain 1) functions in the second step of the beta-oxidation reaction. Multiple studies have demonstrated that ACAT1 can serve as a prognostic marker for ccRCC, with its high expression being associated with better overall survival, which is consistent with our findings (76, 77). The expression product of HPGD (15-hydroxyprostaglandin dehydrogenase) catalyzes a series of dehydrogenation reactions of hydroxylated polyunsaturated fatty acids and is involved in the arachidonic acid metabolism pathway. Furthermore, the risk gene DGKZ (diacylglycerol kinase) participates in the glycerolipid metabolism and glycerophospholipid metabolism pathways. Highly proliferative cancer cells require *de novo* synthesis of fatty acids to sustain the production of glycerophospholipids, especially for membrane production (78). Moreover, DGKZ can act as a central switch between second messenger-activated signaling pathways, negatively regulating T cell receptor signal transduction (79). Subsequently, the machine learning model constructed based on the five prognostic signatures exhibited excellent performance in predicting the occurrence and prognosis of ccRCC in multiple external validation sets. In conclusion, these results confirm the reliability of the five lipid metabolism-related prognostic signatures we identified, indicating their significant potential in ccRCC.

Based on five lipid metabolism-related prognostic signatures, we constructed a lipid metabolism risk score (LMRS) as a risk index in ccRCC. We found that LMRS is an independent prognostic factor and significantly correlates with poor outcomes in ccRCC patients. Moreover, there exists a significant positive correlation between LMRS and tumor mutation burden (TMB). When combined with TMB, LMRS demonstrates improved predictive capability for patient prognosis. TMB serves as a metric for the number of cancer mutations, with higher mutation rates resulting in more neoantigens, thereby increasing the chances of triggering T cell responses and potentially eliciting a response to immune therapy (80). Using deconvolution methods, we assessed the relative infiltration levels of multiple immune cells within the TME in the TCGA-KIRC cohort. We discovered a significant positive correlation between LMRS and the infiltration levels of macrophage M1, T cell CD8+, T cell regulatory (Tregs), myeloid dendritic cell, and macrophage M2, while significant negative correlations were observed with the infiltration levels of NK cell, B cell, and neutrophil. Tumor-infiltrating leukocytes, such as myeloid cells and T cell regulatory (Tregs), can be modulated by malignant tumor cells to evade immune attacks from cytotoxic immune cells at the primary tumor site, enhancing tumor cell survival and promoting the dissemination of tumor cells to metastatic sites (81). T cells represent one of the most abundant and prominently featured cell populations in the TME of solid tumors (82). Additionally, tumor-associated macrophages (TAMs) constitute another crucial immune population within the TME, exerting either inhibitory or promotive effects on tumor growth (83). Consequently, both T cells and TAMs have emerged as promising therapeutic targets and attractive biomarkers (84). Among the multiple subtypes of T cells, CD8+ cytotoxic T cells release various cytolytic mediators, leading to the dissolution and death of target cells, playing a crucial role in the body’s anti-tumor mechanisms (85). We observed a significantly higher infiltration level of T cell CD8+ in the High-LMRS group. The nutritional competition between multiple cellular components within the TME, including glucose and lipid competition, affects the transport, differentiation, and function of T cell CD8+ (86). Indeed, within established tumors, T cells CD8+ are often found to be functionally impaired (87). Our study revealed a significantly higher T cell dysfunction score in the High-LMRS group of ccRCC samples. Additionally, the High-LMRS group exhibited significantly higher stromal score, immune score, and overall ESTIMATE score. These results suggest that the LMRS we established may serve as a risk indicator associated with the immune and metabolic homeostasis within the TME.

In the treatment of ccRCC patients, a combination of immune checkpoint inhibitors (ICIs) and vascular endothelial growth factor receptor tyrosine kinase inhibitors (TKIs) can be used for high-risk disease patients requiring systemic therapy (88). However, there is currently no established standard treatment regimen, and prospective controlled trials are also limited (89). In terms of immunotherapy for ccRCC, some immune checkpoint inhibitors such as nivolumab (anti-PD1) have shown significant clinical benefits for patients with advanced ccRCC (90). Our study revealed extensive positive correlations between LMRS and the risk gene DGKZ with multiple immune checkpoints, including PDCD1 (PD1), CTLA4, and others. Furthermore, the LMRS of samples responding to immunotherapy was significantly higher than that of non-responsive samples, indicating that patients with higher LMRS may be more likely to benefit from immunotherapy. In the realm of anticancer drugs, first-line treatments for metastatic RCC, such as sunitinib and pazopanib, have demonstrated improved overall survival and response rates and prolonged progression-free survival (91, 92). We found a significant negative correlation between LMRS and the IC50 values of sunitinib, axitinib, temsirolimus, rapamycin, and pazopanib, suggesting that patients with higher LMRS are more sensitive to these drugs. Additionally, our analysis of drug therapeutic targets indicated that the High-LMRS group is more sensitive to anticancer drugs targeting kinases and DNA replication, whereas the Low-LMRS group is more sensitive to anticancer drugs targeting RTK signaling and EGFR signaling. Currently, there is still controversy regarding the optimal treatment choices and strategies for individual patients, with nivolumab in combination with cabozantinib showing significant advantages over sunitinib in terms of progression-free survival, overall survival, and the likelihood of remission (93). Furthermore, the combination of lenvatinib and pembrolizumab demonstrated significantly longer progression-free survival and overall survival compared to sunitinib (94). In summary, the LMRS we developed may serve as a significant signature for assessing immunotherapy response and targeted therapy, and we hope it will facilitate the development of precise treatment approaches for ccRCC.

On the whole, our study systematically investigated the association between lipid metabolism and the prognosis of ccRCC patients. We identified five lipid metabolism-related prognostic signatures for ccRCC. Moreover, through machine learning models, we extensively analyzed and validated the potential significance of these prognostic signatures for occurrence and prognosis of ccRCC. Additionally, based on these signatures, we developed a lipid metabolism risk score (LMRS) as a risk index. After our comprehensive analysis, the LMRS we developed may be applied to the risk assessment of ccRCC patients, contributing to clinical practitioners in diagnosing ccRCC patients and providing them with optimal precise treatment plans. However, despite deepening our understanding of the association between lipid metabolism and ccRCC, there are still some limitations. While we retrospectively constructed and validated our findings in public research cohorts, the lack of sufficiently large prospective studies hinders the assessment of the clinical utility of the prognostic signatures we constructed in ccRCC. In conclusion, our findings may have important implications for the diagnosis, prognostic evaluation, and precise treatment of ccRCC.

## Data availability statement

The original contributions presented in the study are included in the article/**Supplementary Material**, further inquiries can be directed to the corresponding author/s.

## Ethics statement

Ethical approval was not required for the studies on humans in accordance with the local legislation and institutional requirements because only commercially available established cell lines were used.

## Author contributions

WC: Writing – original draft, Visualization, Validation, Software, Formal analysis, Data curation. ZZ: Writing – original draft, Validation, Formal analysis. HZ: Writing – original draft, Software. SD: Writing – original draft, Software. XL: Writing – original draft, Validation. SH: Writing – original draft, Validation. SZ: Writing – review & editing, Validation. KC: Writing – review & editing, Data curation.

## Supplementary material

Supplementary Figure 1 Analysis results of key LMDPs in bulk transcriptome. **(A)** Univariate Cox analysis results of key LMDPs. **(B)** Differential expression of key LMDPs between ccRCC samples and normal control samples in TCGA-KIRC cohort. **(C)** Differentially expressed gene results from ANOVA analysis in GEPIA2.

Supplementary Figure 2 The survival analysis results of 5 prognostic signatures from the HPA.

Supplementary Figure 3 Comparison of LMRS among different age **(A)** and gender **(B)** groups.
