# Supplementary figures and images for: Development of prognostic signatures and risk index related to lipid metabolism in ccRCC
Source: Front Oncol. 2024 Jun 13;14:1378095. doi: 10.3389/fonc.2024.1378095 (PMC11208495; doi:10.3389/fonc.2024.1378095)

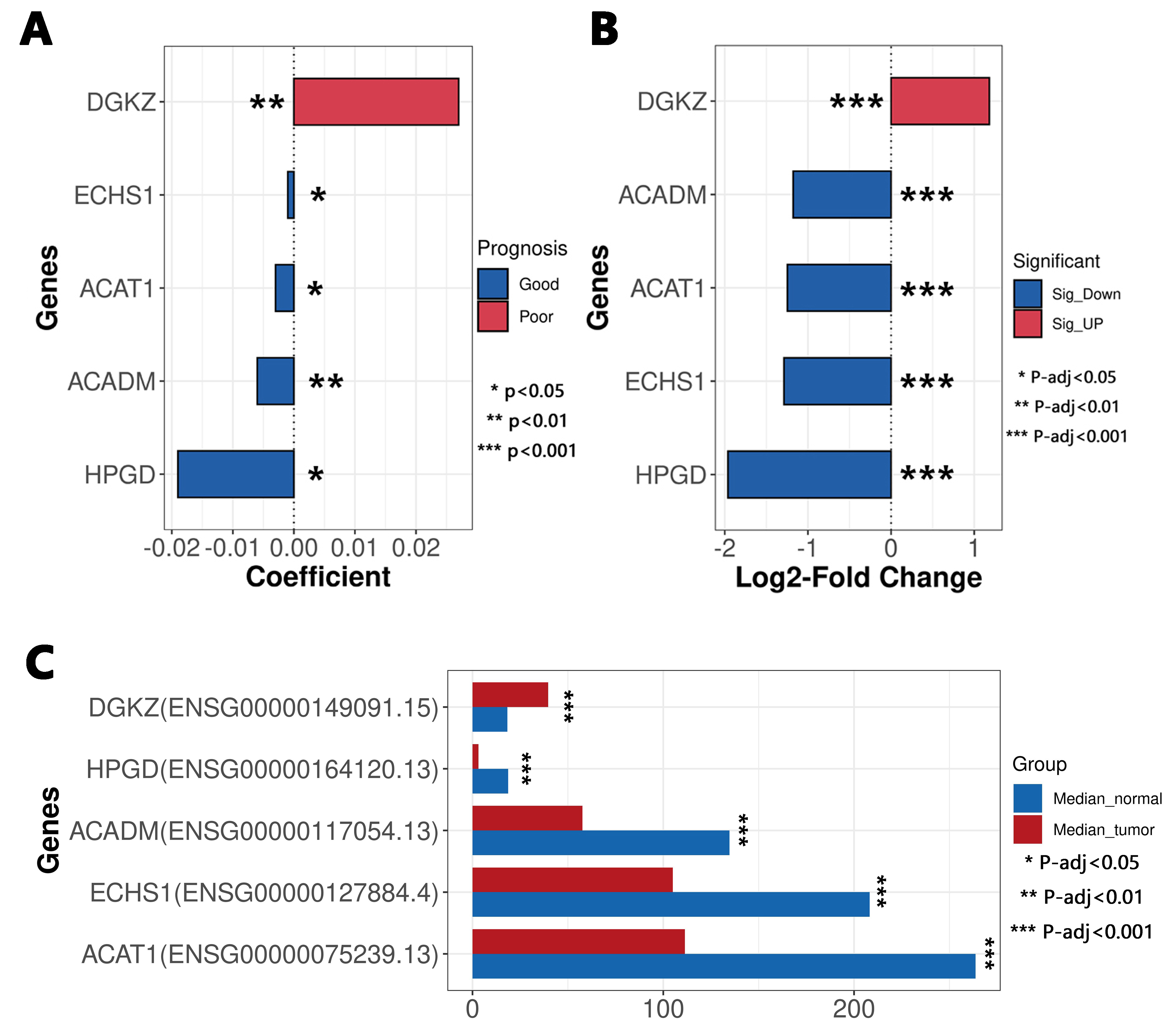

Supplement: Supplementary Figure 1 — Analysis results of key LMDPs in bulk transcriptome. (A) Univariate Cox analysis results of key LMDPs. (B) Differential expression of key LMDPs between ccRCC samples and normal control samples in TCGA-KIRC cohort. (C) Differentially expressed gene results from ANOVA analysis in GEPIA2. [file Image_1.jpeg]

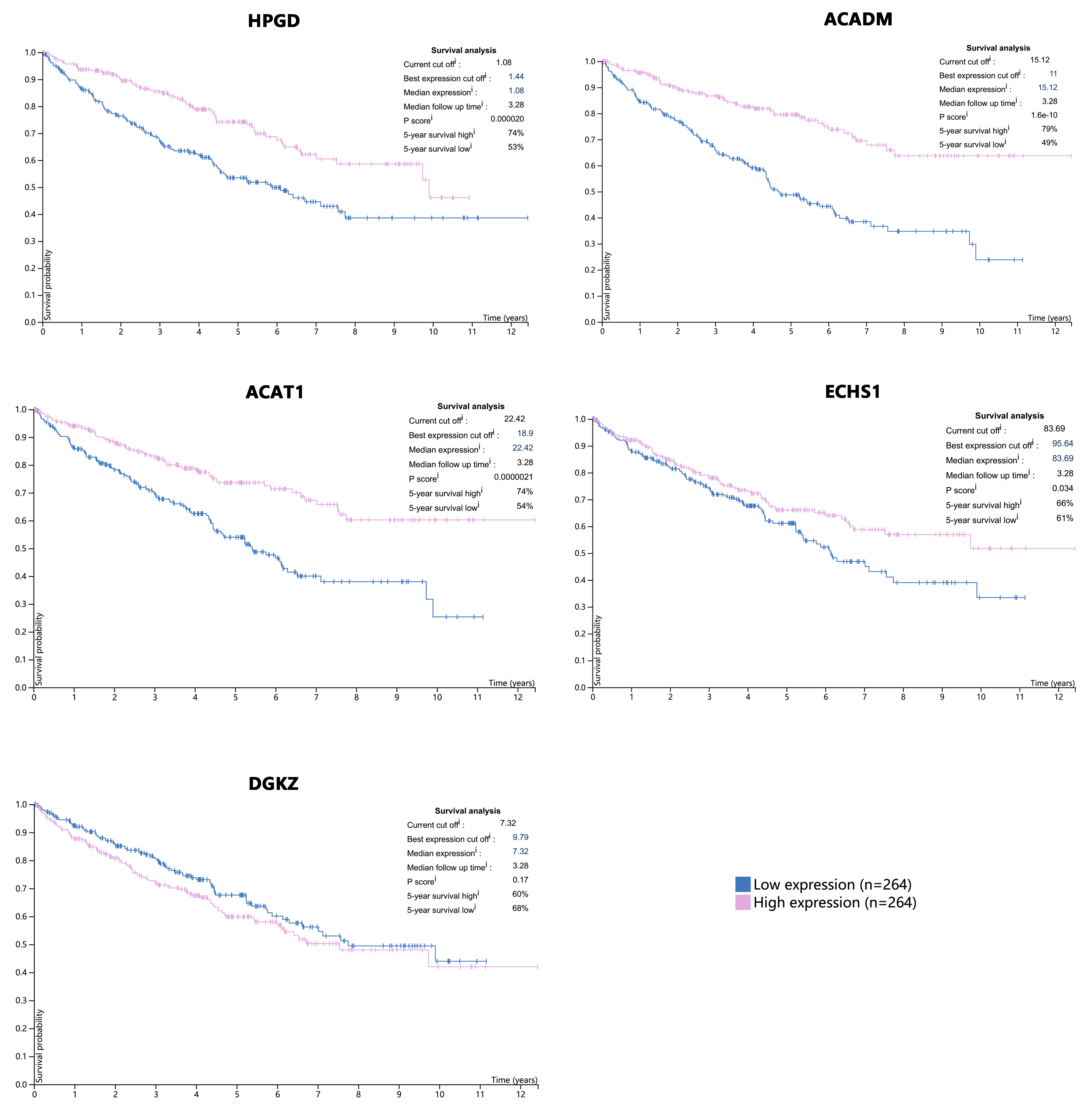

Supplement: Supplementary Figure 2 — The survival analysis results of 5 prognostic signatures from the HPA. [file Image_2.jpeg]

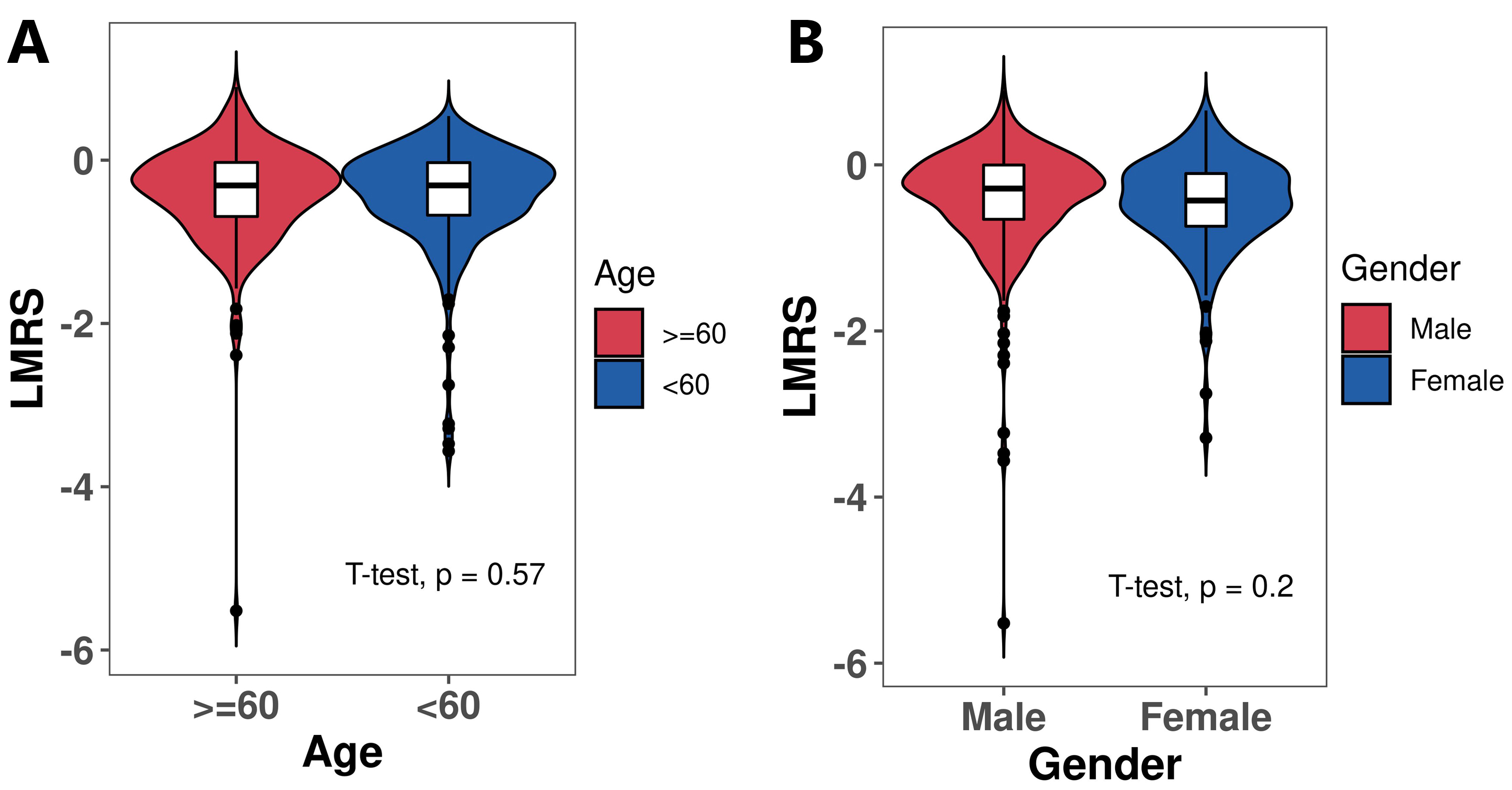

Supplement: Supplementary Figure 3 — Comparison of LMRS among different age (A) and gender (B) groups. [file Image_3.jpeg]
